# Conservative Outcomes of Subtalar Dislocation: A Systematic Review and Meta‐Analysis of the Current Literature

**DOI:** 10.1002/jfa2.70231

**Published:** 2026-10-14

**Authors:** Ahmed Bakr, Yusuf Yusuf, Marwan Tahoun, Anand Pillai

**Affiliations:** ^1^ University of Liverpool School of Medicine Liverpool UK; ^2^ Warrington and Halton Hospitals NHS Foundation Trust Warrington UK; ^3^ Manchester University NHS Foundation Trust Manchester UK

**Keywords:** closed reduction, conservative management, post‐traumatic arthritis, subtalar dislocation, systematic review

## Abstract

**Background:**

Subtalar dislocation is a rare injury accounting for 1%–2% of all dislocations. Although prompt closed reduction is the accepted initial treatment, optimal conservative management protocols remain unclear, and no prior systematic review has synthesised outcomes of purely conservatively managed cases across both medial and lateral dislocations. This review aims to systematically assess the rates of post‐traumatic osteoarthritis, functional outcomes, subtalar stiffness, avascular necrosis, and need for subsequent surgery following conservative management of acute isolated subtalar dislocations.

**Methods:**

A systematic review was conducted according to the PRISMA 2020 guidelines. PubMed, Cochrane Library, CINAHL, Web of Science and OVID were searched for studies reporting outcomes of conservatively managed acute isolated subtalar dislocations. Eligible studies included case series and cohort studies reporting radiographic or functional outcomes following closed reduction and immobilisation. Risk of bias was assessed using the Joanna Briggs Institute (JBI) Critical Appraisal Checklist for case series. Radiographic post‐traumatic arthritis rates were pooled using random‐effects meta‐analysis; functional and other outcomes were synthesised narratively. Given the clinical heterogeneity of the included studies, this review adopted an exploratory descriptive framework.

**Results:**

Ten studies comprising 201 feet were included. All cases were case series (9 retrospective, 1 prospective) with mean follow‐up ranging from 12 to 122 months. Medial dislocations predominated (75%–80%). Pooled radiographic post‐traumatic arthritis occurred in 36% (95% CI: 18%–53%; *I* ^2^ = 43%), though many patients remained functionally well despite structural changes. Functional outcomes were generally favourable for closed medial injuries, with the highest scores achieved using early mobilisation protocols. Reduced subtalar range of motion was common, affecting 42.8%–76.6% of patients where reported. Avascular necrosis was rare (0.5%) and subsequent subtalar fusion was required in only 4.2% of cases. Lateral dislocations had significantly worse outcomes than medial injuries.

**Conclusion:**

Radiographic post‐traumatic arthritis is common following conservative management of subtalar dislocations (36%) but was frequently not associated with functional impairment; favourable functional outcomes were achieved for closed medial injuries, although symptom severity was inconsistently reported across studies. Avascular necrosis is rare. Lateral dislocations consistently have poorer prognoses.

## 1 Introduction

Subtalar dislocation, defined as simultaneous dislocation of the talocalcaneal and talonavicular joints, is a rare but serious injury accounting for 1%–2% of all large‐joint dislocations [1, 2, 3]. Medial dislocations represent approximately 75%–85% of cases, whereas lateral, anterior, and posterior variants are less common but more often associated with high‐energy trauma, soft‐tissue compromise, and poorer functional prognosis [1, 2, 4, 5]. Although uncommon, these injuries can lead to substantial long‐term morbidity, including chronic pain, reduced subtalar range of motion, and post‐traumatic osteoarthritis; talar avascular necrosis, while rare in closed isolated injuries, remains an important concern [2, 4, 5]. Current practice supports prompt closed reduction under anaesthesia and post‐reduction imaging to confirm concentric alignment and exclude associated fractures, followed by cast immobilisation and rehabilitation as the standard approach for most closed medial subtalar dislocations [4, 5, 6]. For lateral, anterior, and posterior dislocations, which are rarer and more frequently associated with high‐energy mechanisms, open wounds, and associated fractures, conservative management is less firmly established as the standard of care, and a lower threshold for surgical intervention is generally adopted.

Despite multiple case series and narrative reviews, guidance on optimal conservative management remains limited [4, 5, 6]. Much of the existing literature pools heterogeneous injuries (medial and lateral dislocations, open and closed trauma, associated fractures) and often combines nonoperative care with transarticular K‐wire fixation or early arthrodesis without reporting outcomes separately for purely conservatively managed cases [1, 2, 3, 4, 5, 6]. Immobilisation duration ranges from 2 to 12 weeks, rehabilitation protocols are poorly defined, and outcome reporting is inconsistent. Critically, no prior systematic review has synthesised outcomes across the full spectrum of acute isolated subtalar dislocations (medial and lateral) managed conservatively; a recent systematic review with individual‐participant‐data analysis addressed only isolated medial dislocations sustained during sporting activities and did not include lateral dislocations or the broader non‐sporting injury population synthesised here [7].

The aim of this review is to systematically assess the rates of post‐traumatic osteoarthritis, functional outcomes, subtalar stiffness, avascular necrosis, and need for subsequent surgery following conservative management of acute isolated subtalar dislocations. We aim to clarify in which scenarios conservative management is appropriate, where caution is warranted, and where current data remain insufficient, thereby informing clinical decision‐making and priorities for future research. Throughout this review, conservative (non‐operative) management denotes closed reduction followed by immobilisation and structured rehabilitation, without surgical (e.g., K‐wire) fixation or primary arthrodesis.

## 2 Methods

This systematic review was conducted and reported in accordance with the PRISMA 2020 statement [8]. The protocol was registered on PROSPERO (ID: CRD420251252126) [9].

### 2.1 Eligibility Criteria

#### 2.1.1 Population

- Patients with acute subtalar/peritalar isolated dislocation
- Diagnosed clinically and/or radiographically

#### 2.1.2 Intervention

- Conservative treatment including closed reduction, immobilisation, and rehabilitation
- Extractable post‐treatment outcomes reported

#### 2.1.3 Comparator

- Pre‐injury functional status (before‐after design). No direct comparison with surgical management was planned or performed within this review.

#### 2.1.4 Outcomes

- Primary: Radiographic post‐traumatic arthritis
- Secondary: functional outcome as reported by study authors, subtalar range of motion, avascular necrosis, need for subsequent surgery, redislocation, pain

#### 2.1.5 Study Design

- Included: randomised trials, prospective or retrospective cohorts, and case series
- Excluded: single‐case reports, reviews, editorials, letters, conference abstracts without full text, and studies not reporting conservative outcomes separately

#### 2.1.6 Language

- Inclusion restricted to English‐language articles

### 2.2 Search Strategy

Five electronic databases were searched: PubMed (MEDLINE), Cochrane Central Register of Controlled Trials (CENTRAL), CINAHL, Web of Science, and OVID. Databases were searched from inception through October 2025. Searches combined free‐text terms and Medical Subject Headings (MeSH) related to the condition and intervention, including ‘subtalar dislocation’, ‘peritalar dislocation’, ‘closed reduction’, ‘conservative treatment’, and ‘treatment outcome’, along with relevant synonyms. All citations were imported into Rayyan for duplicate removal and screening [10].

### 2.3 Study Selection

Titles and abstracts were screened independently by two reviewers using Rayyan software [10]. Studies considered potentially eligible were retrieved in full text and evaluated against inclusion criteria by both reviewers. Disagreements were resolved through consensus discussion. Exclusion reasons at the full‐text stage were documented, and the screening process is presented in the PRISMA 2020 flow diagram (Figure 1) [8].

**FIGURE 1:**
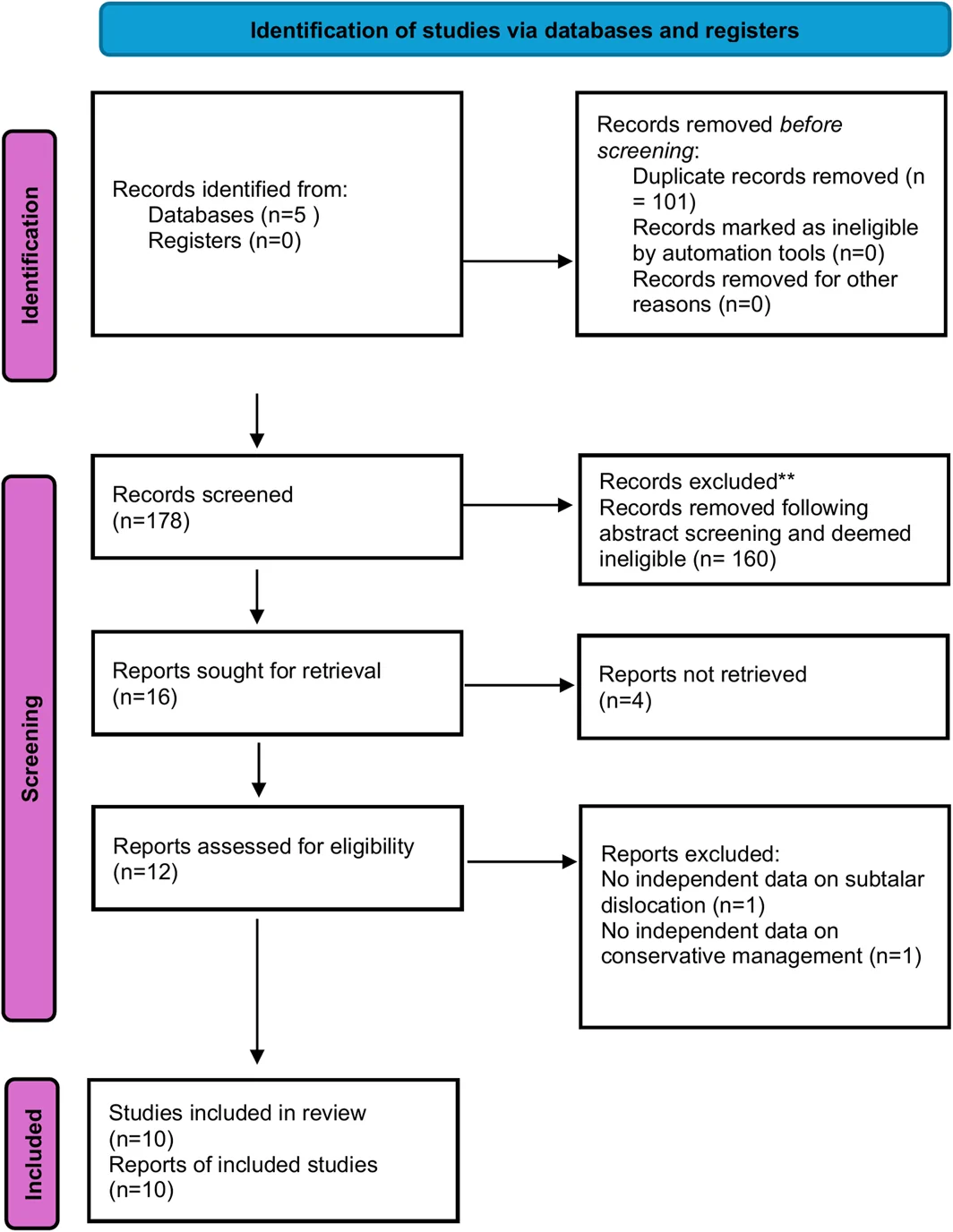
PRISMA‐based summary of the study selection pathway—from database search to the final number of studies included in the review, PRISMA; Preferred Reporting Items for Systematic Reviews and Meta‐Analyses.

Reference lists of the included studies were screened for additional eligible articles. No attempt was made to contact the study authors for unpublished data. Single‐case reports were excluded a priori to reduce the risk of selective outcome reporting and to ensure that outcome estimates derived from series with defined denominators; given the rarity of subtalar dislocation, this exclusion is acknowledged as a potential limitation.

### 2.4 Data Extraction

A standardised data‐extraction form was developed and piloted prior to use. Data were extracted by one reviewer and independently verified by a second reviewer. Extracted variables comprised study identifiers, design, sample size, patient demographics, intervention details, follow‐up duration, radiographic outcomes including post‐traumatic arthritis, functional outcomes (where reported), and complications. No automation tools were employed. Where outcome data were incompletely reported, available data were extracted and the limitations noted; no imputation was performed.

### 2.5 Risk of Bias Assessment

Risk of bias was evaluated using the Joanna Briggs Institute (JBI) Critical Appraisal Checklist for Case Series [11]. Each included study was independently appraised by two reviewers. Responses were categorised as Yes, No, Unclear, or Not Applicable where reporting was insufficient. Discrepancies were resolved by discussion, with referral to a third reviewer if consensus could not be reached. Visual summaries were produced using robvis software (Figures 2 and 3) [12].

**FIGURE 2:**
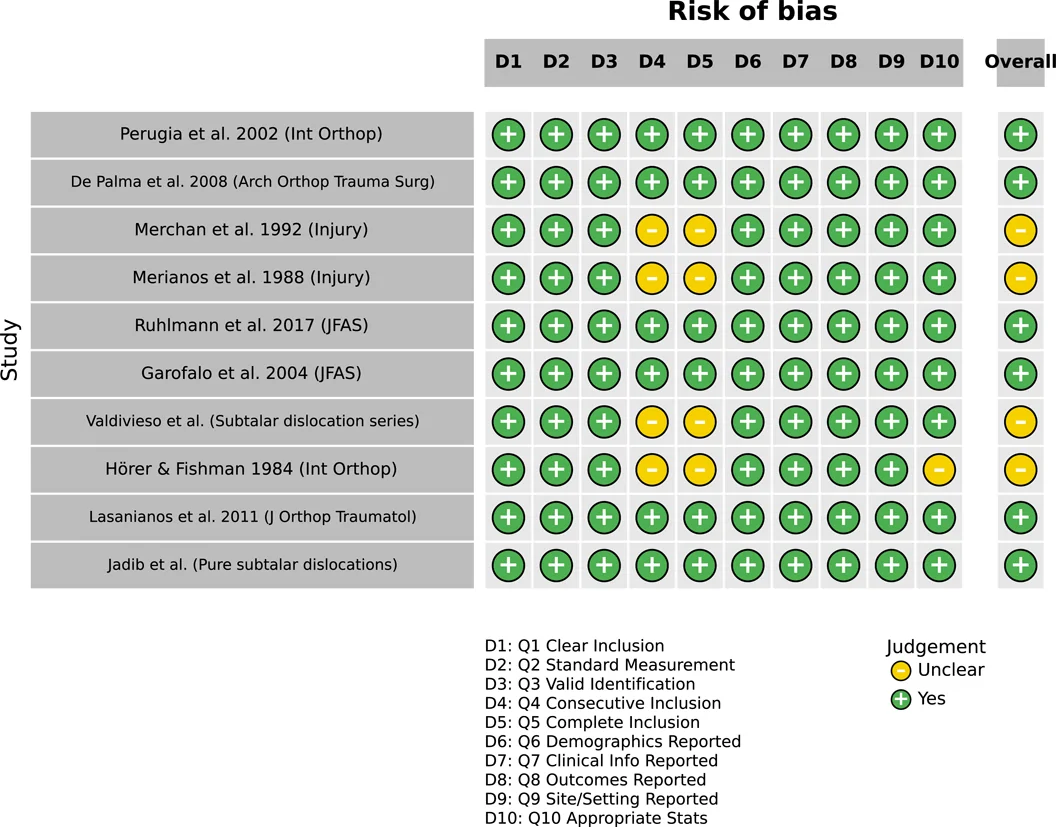
Risk of bias traffic light table of studies used in the review using the JBI tool.

**FIGURE 3:**
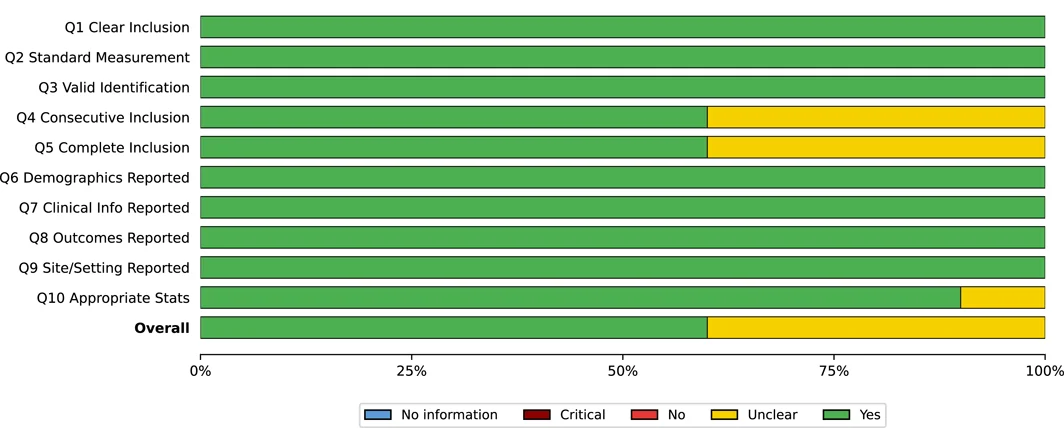
Summary of risk of bias using the JBI tool of studies included in the review.

### 2.6 Data Synthesis

Given the heterogeneity of the included studies, this review adopted an exploratory descriptive framework rather than hypothesis‐testing. Findings were synthesised using a narrative approach. Studies were grouped and compared according to dislocation direction, injury openness, presence of associated fractures, treatment protocol, and follow‐up duration. The primary outcome, radiographic post‐traumatic arthritis, was pooled using a random‐effects model with the Hartung‐Knapp‐Sidik‐Jonkman method using Review Manager (RevMan version 5.4, Cochrane Collaboration). The decision to pool this outcome was based on consistent binary outcome reporting across studies using comparable definitions. Functional outcomes were synthesised narratively due to substantial heterogeneity in outcome measures across studies, which precluded meaningful pooling. Other secondary outcomes, including subtalar range of motion, avascular necrosis, and need for subsequent surgery, were described narratively. Avascular necrosis and need for subsequent surgery were reported too infrequently, and with differing denominators, to justify formal random‐effects meta‐analysis; these outcomes were therefore summarised as simple pooled proportions with accompanying 95% confidence intervals. Statistical analyses were performed by the review authors (A.B. and Y.Y.), with methodological oversight from the senior authors (M.T. and A.P.); no independent biostatistician was involved. A formal sensitivity analysis (e.g., excluding studies published before 2000) was not feasible given the small number of pooled studies. The certainty of evidence for the primary outcome was appraised using the GRADE approach; as the evidence derived entirely from non‐comparative case series, certainty began at low and was rated very low after downgrading for risk of bias and imprecision.

### 2.7 Publication Bias

Formal assessment of publication bias (e.g., funnel‐plot asymmetry) was not undertaken because fewer than 10 studies contributed to the pooled analysis, below the threshold at which such tests are sufficiently powered and interpretable, in line with Cochrane guidance.

## 3 Results

We identified 279 studies through database searches. After removing 101 duplicates, 178 papers remained for title and abstract screening. Following this, 16 papers underwent full‐text screening. Four papers were excluded as full texts were unavailable, one was excluded due to lack of independent data on subtalar dislocations, and one was excluded due to lack of independent data on conservative outcomes. Ultimately, 10 studies were included in the review.

### 3.1 Narrative Synthesis

Across the 10 included studies, 201 feet with acute isolated subtalar dislocations were managed conservatively [13, 14, 15, 16, 17, 18, 19, 20, 21, 22]. All studies were retrospective case series except Lasanianos et al., which was prospective. Medial dislocations predominated (75%–80%), consistent with the epidemiological literature [1, 2]. Mean follow‐up ranged from 12 to 122 months. The characteristics of the included studies are summarised in Table 1, and their outcomes in Table 2.

**TABLE 1:** Characteristics of included studies.

Study	Study design	*n* (feet)	Injury type	Intervention	Mean age (years)	Mean follow‐up (months)
Perugia et al. (2002) [13]	Retrospective case series	45	37 medial, 8 lateral; all closed	Closed reduction, 4 weeks cast immobilisation	NR	89
De Palma et al. (2008) [14]	Retrospective case series	30	20 medial, 10 lateral; 6 open	Closed reduction under GA, 35 days cast (6 weeks if fracture)	33	101
Merchan et al. (1992) [15]	Retrospective case series	39	29 medial, 10 lateral; 16 open (41%); 25 with fractures	Closed reduction under GA, mean 4.7 weeks cast	35.5	66
Merianos et al. (1988) [16]	Retrospective case series	21	18 medial, 3 lateral; all closed	Closed reduction under GA, 10–12 weeks cast	31.8	76
Ruhlmann et al. 2017 [17]	Retrospective case series	13	10 medial, 3 lateral; 1 open	Closed reduction under GA, 6 weeks NWB, then physiotherapy and progressive weight‐bearing	38.5	72.6
Garofalo et al. (2004) [18]	Retrospective case series	18	13 medial, 5 lateral; 6 open; 7 with fractures	Closed reduction under GA, 4 weeks cast (6 weeks if fracture)	34	122
Valdivieso et al. (1996) [19]	Retrospective case series	10	All medial; all closed	Closed reduction under GA, mean 4.7 weeks cast	31.7	95
Hörer & Fishman (1984) [20]	Retrospective case series	10	All medial; all closed	Closed reduction under GA, 10–12 weeks cast	NR (18–61)	12
Lasanianos et al. (2011) [21]	Prospective case series	8	All medial; all closed	Closed reduction under sedation, 2 weeks cast then early mobilisation	37.25	36
Jadib et al. (2024) [22]	Retrospective case series	7	6 medial, 1 lateral; all closed	Closed reduction under GA, 6 weeks NWB cast	29.14	38.2

Abbreviations: GA, general anaesthesia; NR, not reported; NWB, non‐weightbearing.

**TABLE 2:** Summary of outcomes across included studies.

Study	Functional outcome	Subtalar ROM	Post‐traumatic arthritis	Complications/Notes
Perugia et al. (2002) [13]	Mean AOFAS 83.8 (± 19.2); 12/45 scored 100; 27% recreational limitation	NR	Moderate‐severe in 12/45 (27%)	1/45 required subtalar fusion; no AVN reported
De Palma et al. (2008) [14]	Mean AOFAS 78.8 (± 19.2); 7 excellent (all medial), 14 good, 6 fair, 3 poor	Reduced in 76.6%	Present in 46.7%	3/30 required fusion (all lateral); medial AOFAS 87.1 versus lateral 59.3 (*P* = 0.05)
Merchan et al. (1992) [15]	11 good (28%), 7 fair (18%), 21 poor (54%) by hardcastle criteria	Frequently reduced	Common	1 AVN; 10 reflex sympathetic dystrophy; all open injuries fair/poor
Merianos et al. (1988) [16]	7/17 medial occasional pain; 3/17 chronic pain; all 3 lateral severe chronic pain	10/20 limited motion	9/20 (45%)	2/3 lateral required arthrodesis; 2 tibial nerve dysesthesia
Ruhlmann et al. (2017) [17]	Mean AOFAS 80.1 (66–90); 9/13 good (69%), 4/13 poor (31%)	Reduced in 61.5%	Present in 46.2%	No AVN; 6 weeks NWB immobilisation
Garofalo et al. (2004) [18]	10/18 excellent (56%, all closed medial), 3 fair (17%), 5 poor (28%)	45% had reduced ROM	Present in fair/poor cases	No AVN; lateral and open injuries had worse outcomes
Valdivieso et al. (1996) [19]	6 good (60%), 4 fair (40%)	Impaired in fair cases	Present in fair cases	No AVN; no poor outcomes in conservative subgroup
Hörer & Fishman (1984) [20]	All 10 patients had normal pain‐free gait at 1 year	NR	None at 1 year	No AVN; short follow‐up (12 months)
Lasanianos et al. (2011) [21]	Mean AOFAS 90.75 (82–97); all returned to activities at ∼2 months	92.5% of contralateral	None at final follow‐up	No AVN; early mobilisation protocol (2 weeks cast)
Jadib et al. (2024) [22]	Mean AOFAS 83 (71–91); 1 excellent, 5 good	Reduced in 42.8%	1/7 (14.3%)—the lateral case	No AVN; emergent reduction within 4 hours

Abbreviations: AOFAS, american orthopaedic foot and ankle society ankle‐hindfoot Score; AVN, avascular necrosis; ROM, range of motion; NR, not reported.

Functional outcomes were favourable for closed medial injuries, with the highest scores achieved in cohorts using early mobilisation protocols [13, 14, 17, 21, 22]. Several studies employed the AOFAS Ankle‐Hindfoot Score, which, although it remains one of the most widely used instruments in foot and ankle research, has been shown to have limited psychometric validation as a primary outcome measure [23]. Functional results are therefore described in terms of return to activity and pain rather than reliance on any single instrument. De Palma et al. demonstrated significantly better outcomes for medial versus lateral dislocations (*P* = 0.05) [14]. Outcomes were consistently poorer for lateral dislocations, open injuries, and fracture‐associated cases, with all three fusions in De Palma et al. occurring in lateral injuries, and Merianos et al. reporting severe chronic pain in all lateral cases [14, 16].

Regarding the primary outcome, a pooled meta‐analysis of five studies (115 feet) found radiographic post‐traumatic arthritis in 36% of patients (95% CI: 18%–53%; *I* ^2^ = 43%; Figure 4), pooled across predominantly medial but directionally mixed cohorts and not stratified by dislocation direction, as only one study reported direction‐specific radiographic outcomes. This pooled estimate should be interpreted with caution given the small number of studies, moderate heterogeneity, and the observational nature of the included evidence. Nonetheless, many remained functionally well despite structural changes [13, 14, 16, 17, 22]. Reduced subtalar range of motion was common (42.8%–76.6% where reported) [14, 17, 18, 22]. Avascular necrosis was rare, occurring in only one of 201 feet (0.5%; 95% CI: 0.09%–2.76%); this single case (Merchan et al.) arose in an open medial dislocation with an associated navicular fracture and resolved with spontaneous revascularisation, underscoring the rarity of avascular necrosis after closed isolated injuries. Subsequent subtalar fusion was required in only six of 144 feet (4.2%; 95% CI: 1.5%–8.9%), five of the six fusions following lateral dislocations (three in De Palma et al. and two in Merianos et al.), across 8 studies reporting this outcome [13, 14, 16, 17, 19, 20, 21, 22]. No cases of recurrent instability have been reported in studies using early mobilisation protocols [21].

**FIGURE 4:**
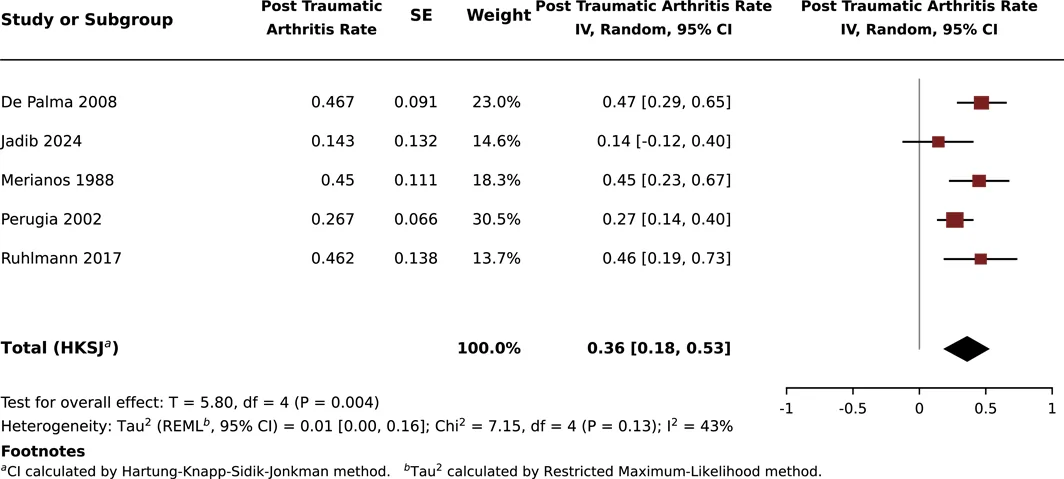
Forest plot of pooled post‐traumatic arthritis rate. SE, Standard Error; CI, Confidence Interval; HKSJ, Hartung‐Knapp‐Sidik‐Jonkman method; and REML, restricted‐Likelihood method.

### 3.2 Risk of Bias

Across the 10 included case series, reporting quality was mainly to a high standard. Most studies clearly defined subtalar dislocation, specified inclusion criteria, described demographics and injury characteristics, and detailed conservative management and follow‐up outcomes. Validated functional outcome tools were frequently used and complications were usually documented, supporting predominantly low risk of bias across domains related to case identification, measurement, and outcome reporting.

Uncertainty arose mainly around cohort selection and analytical transparency. In several studies (e.g., Merchan, Merianos, Hörer) [15, 16, 20], it was unclear whether recruitment was consecutive or complete, and statistical handling was only briefly described, yielding ‘unclear’ judgements for these domains and in the overall rating for 4/10 studies [15, 16, 19, 20]. No study performed a priori power calculations or employed validated patient‐reported outcome measures, and no study reported return‐to‐work or return‐to‐sport data, limiting the assessment of outcomes most meaningful to patients. These issues suggest potential, though unproven, selection and reporting bias. Future research should use prospective, clearly consecutive cohorts, pre‐registered protocols, and standardised outcome sets to strengthen validity and comparability across studies.

## 4 Discussion

### 4.1 Principal Findings

This systematic review synthesised outcomes from 10 studies involving 201 feet with acute isolated subtalar dislocations managed conservatively [13, 14, 15, 16, 17, 18, 19, 20, 21, 22]. Regarding the primary outcome, a pooled meta‐analysis demonstrated radiographic post‐traumatic arthritis in 36% of patients (95% CI: 18%–53%; *I* ^2^ = 43%, indicating moderate heterogeneity). This estimate should be regarded as indicative rather than definitive given the small number of pooled studies and the observational nature of the evidence. A key finding was that many remained functionally well despite these structural changes. For closed, medial, fracture‐free injuries, nonoperative management with prompt closed reduction, short‐term immobilisation, and structured rehabilitation results in favourable long‐term functional outcomes [13, 14, 17, 21, 22]. In the largest exclusively conservative cohort, Perugia et al. (*n* = 45) reported favourable outcomes at 7.4 years of follow‐up, with only one patient requiring subsequent fusion [13]. These outcomes were achieved with a low rate of avascular necrosis (1/201 feet) and no reported cases of recurrent dislocation when early mobilisation protocols were used [13, 14, 15, 17, 21].

The evidence consistently indicates that the direction of dislocation is a critical prognostic factor. De Palma et al. reported significantly better outcomes in medial compared with lateral dislocations (*P* = 0.05), with all three patients requiring subsequent subtalar fusion having sustained lateral injuries [14]. A comparative meta‐analysis of medial versus lateral dislocations was not feasible, as only one study reported functional outcomes stratified by dislocation direction. Merianos et al. observed that all three patients with lateral dislocations developed severe chronic pain and antalgic gait, with two requiring arthrodesis, leading to the suggestion that early subtalar fusion may be preferable for lateral injuries [16]. This pronounced prognostic divergence likely reflects the higher energy mechanisms typically responsible for lateral dislocations, which are associated with greater soft tissue disruption and cartilage damage [14, 16, 18].

The consistently poor outcomes observed in lateral dislocations warrant specific consideration. Lateral subtalar dislocations are typically produced by higher‐energy mechanisms involving forced eversion, resulting in greater ligamentous disruption, a higher incidence of open wounds, and more frequent association with additional structural damage to both bone and soft tissue [1, 2, 14, 16]. These features explain why a greater proportion of lateral injuries ultimately require surgical intervention rather than purely conservative management. In the present review, all patients requiring subsequent arthrodesis in De Palma et al. had sustained lateral injuries [14], and all lateral cases in Merianos et al. developed severe chronic pain [16]. Given these findings, early surgical consultation may be warranted for lateral subtalar dislocations, even when closed reduction is achievable, and patients should be counselled regarding the substantially poorer prognosis compared with medial injuries. Importantly, this poorer lateral prognosis does not appear attributable solely to associated open or fracture injuries: in Ruhlmann et al.’s series of exclusively isolated closed dislocations, lateral injuries still achieved lower AOFAS scores than medial injuries (75.3 vs. 82.1), although this difference did not reach statistical significance [17].

In the minority of patients who develop persistent, disabling symptoms, most often following lateral injuries with established subtalar arthrosis, salvage subtalar or hindfoot arthrodesis remains an effective option for pain relief and functional improvement. In the present pooled cohort, subsequent fusion was required in 4.2% of feet and followed lateral dislocations in five of six cases, consistent with arthrodesis serving as a definitive salvage procedure for symptomatic post‐traumatic arthrosis rather than as a primary treatment. Early counselling regarding this possibility is appropriate for higher‐risk (lateral, open, or fracture‐associated) injuries.

In the present review, the best functional outcomes and motion preservation were consistently achieved with shorter immobilisation protocols. The optimal duration of immobilisation remains a key clinical question with evolving evidence. Earlier studies in this review utilised longer immobilisation periods (6–12 weeks) [15, 16, 20], whereas more recent protocols recommend shorter casting durations (2–4 weeks) followed by early supervised mobilisation [13, 21]. The theoretical rationale for early mobilisation was articulated by McKeever in 1963, who argued that the inherent stability of subtalar reduction makes early mobilisation both safe and beneficial for preventing fibrosis [24]. Perugia et al. reported that, in a predominantly medial, all‐closed cohort, 71% of patients experienced some limitation of subtalar movement even after only 4 weeks of immobilisation, referencing Buckingham's finding that most patients immobilised for 6 weeks developed movement limitation and Heppenstall's observation that 80% developed significant subtalar restriction, with 30% developing severe arthritis [25, 26]. Notably, Lasanianos et al. achieved excellent functional outcomes using the shortest immobilisation protocol (approximately 2 weeks) combined with early range‐of‐motion exercises; in that protocol, partial weight‐bearing began from the third week and progressed to full weight‐bearing by the fifth week, assisted by a below‐knee functional brace that permitted plantar‐ and dorsiflexion while restricting inversion and eversion, without increased rates of redislocation or instability [21]. However, Valdivieso et al. cautioned that loss of subtalar mobility following prolonged immobilisation may be preferable to instability resulting from premature mobilisation, indicating that protocols should be individualised based on patient factors and injury characteristics [19].

Subtalar range of motion was reduced in a substantial proportion of patients across studies, ranging from 42.8% to 76.6% [14, 17, 18, 22]. However, studies employing early mobilisation reported better preservation of motion. Lasanianos et al., using an approximately 2‐week immobilisation protocol followed by early range‐of‐motion exercises, achieved a mean subtalar range of motion of 92.5% of the contralateral side with no radiographic arthritis or avascular necrosis [21]. In contrast, Ruhlmann et al., using 6 weeks of non‐weight‐bearing immobilisation, reported reduced subtalar mobility in 61.5% of patients [17]. This supports the notion that prolonged immobilisation contributes to fibrosis and stiffness, whereas early controlled mobilisation may optimise range of motion recovery without compromising stability.

Despite these range of motion deficits, functional recovery was rarely prevented [13, 14, 17, 22]. Regarding the high rate of radiographic post‐traumatic arthritis (36%), a consistent finding was the dissociation between structural changes and symptomatic outcomes (Figure 4) [13, 14, 16, 17, 22]. Garofalo et al., reporting on a mixed cohort of medial and lateral injuries, noted that the subtalar joint is susceptible to arthrosis after dislocation due to compressive and shearing forces during displacement, even in the absence of visible fractures, which may account for arthritis development in patients without associated bony injury [18]. This imaging‐symptom mismatch has important implications for patient counselling: radiographic osteoarthritis should be expected but does not necessarily predict functional disability.

### 4.2 Comparison With Existing Literature

The findings of this review are consistent with the broader subtalar dislocation literature. The predominance of medial dislocations (75%–85%) and the prognostic distinction between medial and lateral injuries align with classic descriptions by DeLee, Curtis, Main and Jowett, who established that lateral dislocations are associated with higher‐energy trauma, greater soft tissue compromise, and poorer functional outcomes [1, 2]. The functional results in this review for closed isolated injuries are comparable to outcomes reported in mixed surgical‐conservative series, suggesting satisfactory outcomes can be achieved with both approaches in appropriately selected patients [4, 5]. These findings are also concordant with more recent focused reports: Biz et al. described favourable outcomes following conservative management of isolated medial subtalar dislocations in athletes [27], and a subsequent systematic review with individual‐participant‐data analysis similarly concluded that conservative treatment of isolated medial subtalar dislocation yields generally favourable functional results, while noting the persistent risk of post‐traumatic subtalar osteoarthritis [7].

The importance of immediate reduction, highlighted in all included studies, is corroborated by vascular anatomy research demonstrating the precarious blood supply to the talus [4, 5, 20]. Hörer and Fishman observed that, although Watson‐Jones reported a 50% incidence of avascular necrosis following peritalar dislocation in 1955 [28], a figure that likely reflects delayed reductions in an era before modern emergency protocols, their series with prompt reduction had no cases, attributing this outcome to early realignment of vessels in surrounding tissues [20]. The rarity of avascular necrosis in this review (1/201 feet) further supports this principle and indicates that the historically feared complication of talar necrosis is largely preventable with timely reduction [13, 14, 15, 20].

The high prevalence of radiographic post‐traumatic osteoarthritis despite satisfactory functional outcomes is consistent with findings in other periarticular trauma literature. Similar dissociations between structural and symptomatic outcomes have been observed following ankle fractures and tibial plateau injuries [4, 5]. Garofalo et al. proposed that the high forces involved in dislocation create cartilaginous lesions not detected on radiographs, which affect long‐term clinical outcomes and may explain the development of arthritis even in the absence of associated fractures [18]. This mechanistic insight suggests that cartilage injury is intrinsic to the dislocation event itself, regardless of subsequent management [15, 16, 17, 18].

### 4.3 Strengths and Limitations

This review has several methodological strengths. It was designed and reported according to PRISMA 2020 guidelines [8], with a protocol registered on PROSPERO prior to data extraction [9]. The comprehensive multi‐database search, dual‐independent screening, and standardised data extraction anchored on pre‐specified outcomes enhance transparency and reproducibility. By focusing specifically on conservatively managed isolated subtalar dislocations, we addressed a gap in the existing literature, which has predominantly pooled heterogeneous injury patterns and treatment strategies [1, 2, 3, 4, 5, 6]. The extraction of injury characteristics (direction, openness, associated pathology) alongside functional outcomes enables meaningful interpretation of prognostic factors [13, 14, 15, 16, 17, 18, 19, 20, 21, 22].

Several limitations should be noted. First, the evidence base is entirely observational, consisting mainly of small, single‐centre, retrospective case series spanning four decades (1984–2024), with inherent risks of selection and reporting bias [13, 14, 15, 16, 17, 18, 19, 20, 21, 22]. Second, outcome measures were not standardised across studies, with various functional scoring systems and non‐validated categorical systems used, precluding direct comparison [13, 14, 15, 16, 17, 18, 19, 22]. No study reported validated patient‐reported outcome measures or return‐to‐work/sport data, limiting the assessment of outcomes most meaningful to patients. Third, substantial clinical heterogeneity exists in injury populations, immobilisation protocols (2–12 weeks), rehabilitation approaches, and follow‐up duration (12–122 months) [13, 14, 15, 16, 17, 18, 19, 20, 21, 22]. Moreover, older studies predating routine CT imaging may have underreported associated osteochondral injuries [15, 16, 20]. Furthermore, inclusion was restricted to English‐language articles, which may have introduced language bias. Several studies included cohorts comprised a mixture of isolated and non‐isolated injuries (open dislocations and associated fractures; e.g., Merchan et al., De Palma et al., and Garofalo et al.), and outcomes for purely isolated closed dislocations could not always be disaggregated from the source data. Finally, confounding by indication is unavoidable in observational data, as injury severity likely influenced both treatment decisions and outcomes.

### 4.4 Clinical Implications

Based on the findings of this review, we propose that closed, medial, fracture‐free subtalar dislocations may be considered for conservative management as first‐line treatment [13, 14, 15, 16, 17, 18, 19, 20, 21, 22]. For isolated closed lateral dislocations, prompt closed reduction followed by conservative management remains a reasonable first‐line approach, since operative treatment is principally reserved for irreducible, open, or fracture‐associated injuries; however, given the consistently poorer outcomes of lateral injuries, these patients warrant early surgical consultation, a lower threshold for operative intervention, and explicit counselling regarding prognosis. Open injuries and fracture‐associated cases, irrespective of dislocation direction, warrant early surgical involvement [14, 16, 18]. Regardless of the injury pattern, urgent closed reduction should be performed as soon as possible, ideally under adequate anaesthesia or sedation to achieve muscle relaxation and minimise articular trauma from multiple reduction attempts [4, 5, 17, 22]. Reduction should be performed as expeditiously as possible, though no specific time threshold has been validated in the literature; delays increase soft tissue swelling and reduction difficulty. The urgency of reduction is important not only for skin viability over the prominent talar head but also for preservation of the tenuous talar blood supply [17, 20, 22]. Post‐reduction imaging, preferably CT, should confirm concentric alignment and exclude occult osteochondral fragments that may influence rehabilitation pacing [4, 5, 17, 21, 22].

The available evidence suggests shorter immobilisation periods (2–4 weeks) followed by early supervised mobilisation for uncomplicated medial dislocations [13, 21]. This approach may optimise functional recovery by preventing the fibrosis and stiffness associated with prolonged casting, without compromising stability [20]. Protocols should be individualised based on patient factors, injury energy, and any associated pathology [13, 17, 19].

Patient counselling should address the expected trade‐offs of conservative management. Even after successful treatment, some degree of subtalar stiffness is anticipated in most patients, although this rarely prevents return to pre‐injury activities [13, 14, 17, 21, 22]. Radiographic osteoarthritis should be expected but does not necessarily predict functional disability, given the consistent imaging‐symptom mismatch observed across studies [13, 14, 15, 17, 18]. Patients should be informed that activity‐related symptoms may develop or progress over time, warranting long‐term surveillance even after apparently successful early outcomes [13, 14, 18]. These recommendations are derived from observational data of limited quality and should be regarded as provisional pending prospective validation.

### 4.5 Future Research Priorities

The limitations identified in this review highlight clear priorities for future research. First, prospective multicentre cohort studies with standardised outcome assessment are needed [13, 14, 15, 16, 17, 18, 19, 20, 21, 22]. A core outcome set should include validated functional outcome measures for comparability with existing literature [13, 14, 17, 21, 22], validated patient‐reported outcome measures (such as the Foot Function Index or FAAM), subtalar range of motion measured by standardised goniometric method, and uniform radiographic osteoarthritis grading (such as Kellgren‐Lawrence adapted for the subtalar joint). Studies should prospectively stratify and report outcomes by dislocation direction, injury mechanism, openness, and associated fracture burden [1, 2, 3, 4, 5, 6, 13, 14, 15, 16, 17, 18, 19, 20, 21, 22].

Comparative effectiveness studies examining conservative versus surgical management for specific injury subtypes (e.g., lateral dislocations, fracture‐associated cases) would help clarify optimal treatment selection. Registry‐based approaches may facilitate the recruitment of sufficient case numbers for meaningful comparison given the rarity of this injury.

Pragmatic comparative studies evaluating immobilisation duration and rehabilitation protocols are also warranted, addressing questions such as optimal casting duration (2–4 vs. 6 weeks), timing of weightbearing progression, and intensity of physiotherapy [13, 17, 21]. Outcomes should include return to work and sport alongside functional scores.

Additionally, the role of advanced imaging in guiding management and predicting prognosis requires further investigation [17, 21, 22]. MRI assessment of cartilage integrity at presentation may help identify patients at higher risk of post‐traumatic arthritis, potentially informing counselling and surveillance protocols [17]. Similarly, the predictive value of specific CT findings (joint congruence, occult osteochondral lesions) for functional outcomes remains unclear [4, 5, 17, 22].

Finally, partial subtalar ligament injuries, particularly isolated lateral talocalcaneal ligament (LTCL) damage, sometimes in conjunction with calcaneocuboid or lateral ankle ligament injuries, likely represent an underdiagnosed spectrum of subtalar instability. Further investigation of these less frequently diagnosed ligamentous injuries may improve understanding of the full continuum of subtalar pathology, from partial ligamentous sprains through to complete dislocation.

## 5 Conclusion

Radiographic post‐traumatic arthritis is common following conservative management of subtalar dislocations (36%) but frequently occurs without corresponding functional impairment, with favourable functional outcomes achieved for closed medial injuries and low rates of avascular necrosis. Subtalar stiffness is frequent but rarely prevents return to activities. Lateral dislocations, open injuries, and fracture‐associated cases have consistently poorer prognoses. Future research should prioritise prospective studies with standardised outcomes to clarify optimal immobilisation duration and rehabilitation protocols.

## Author Contributions

**Ahmed Bakr:** conceptualization, data curation, formal analysis, investigation, methodology, project administration, validation, visualization, writing – original draft, writing – review and editing. **Yusuf Yusuf:** conceptualization, data curation, formal analysis, investigation, methodology, validation, visualization, writing – original draft, **Marwan Tahoun:** methodology, supervision, writing – review and editing, **Anand Pillai:** methodology, supervision, writing – review and editing.

## Funding

The authors have nothing to report.

## Ethics Statement

The authors have nothing to report.

## Consent

The authors have nothing to report.

## Conflicts of Interest

The authors declare no conflicts of interest.

## Data Availability

All data generated or analysed during this study are included in this published article and its tables. The search strategies and data extraction forms are available from the corresponding author on reasonable request. The review protocol is registered on PROSPERO (CRD420251252126).
